# Beta-Meta: a meta-analysis application considering heterogeneity among genome-wide association studies

**DOI:** 10.5808/gi.22046

**Published:** 2022-12-30

**Authors:** Gyungbu Kim, Yoonsuk Lee, Jeong Ho Park, Dongmin Kim, Wonseok Lee

**Affiliations:** Medical Genomics R&D, JLK Inc., Seoul 06141, Korea

**Keywords:** genome-wide association studies, heterogeneity, meta-analysis, python application, single nucleotide polymorphism

## Abstract

Many packages for a meta-analysis of genome-wide association studies (GWAS) have been developed to discover genetic variants. Although variations across studies must be considered, there are not many currently-accessible packages that estimate between-study heterogeneity. Thus, we propose a python based application called Beta-Meta which can easily process a meta-analysis by automatically selecting between a fixed effects and a random effects model based on heterogeneity. Beta-Meta implements flexible input data manipulation to allow multiple meta-analyses of different genotype-phenotype associations in a single process. It provides a step-by-step meta-analysis of GWAS for each association in the following order: heterogeneity test, two different calculations of an effect size and a p-value based on heterogeneity, and the Benjamini-Hochberg p-value adjustment. These methods enable users to validate the results of individual studies with greater statistical power and better estimation precision. We elaborate on these and illustrate them with examples from several studies of infertility-related disorders.

## Introduction

Genome-wide association studies (GWAS) of diseases and traits have increasingly been used to identify single nucleotide polymorphisms (SNPs). Although GWAS have tested hundreds of thousands of genetic variants to discover genotype-phenotype associations, they have a few limitations. Variants discovered in individual GWAS explain only a small proportion of heritability, and their genetic effect sizes are mostly small and require a substantial sample size to identify [1,2]. Moreover, some studies examining the same genotype-phenotype association yield inconsistent results such as variant effect sizes in opposite directions [3,4]. To overcome these limitations, a meta-analysis of GWAS has been used extensively since it can improve the statistical power by combining data across any number of independent studies and can clarify heterogeneity among their results [5].

As meta-analysis has become a popular tool for aggregating data from multiple sources, several studies have revised analytical strategies from previous well-known studies [6-9]. A weighted average of the effect sizes can be calculated under a fixed effects model or a random effects model, but the fixed effects model can lead to false-positive results when there is heterogeneity between studies [9,10]. Even though it is important to use the appropriate approaches for meta-analyses, there are few available tools that provide a step-by-step calculation, running both the fixed effects model and the random effects model [10]. Therefore, for those who find it difficult to conduct a meta-analysis, we have developed a flexible data processing tool that adopts the revised methods assessing heterogeneity between studies and using the Benjamini-Hochberg (BH) procedure to calculate adjusted p-values [11]. In addition to these methods, Beta-Meta has several convenient features such as an automatic selection between the two models depending upon the quantified heterogeneity. It also manifests flexibility and convenience in processing data as it can perform a varying number of meta-analyses simultaneously and operate strand flipping automatically when there is a discrepancy in the direction of the strand orientation between studies. Also, we have attached haploR package [12] which detects alternative SNPs by estimating their correlations.

Since it is crucial to increase statistical power in order to identify significant variants, especially in studies with small sample sizes, we demonstrate Beta-Meta using studies of diseases related to infertility, most of which have relatively small sample sizes [4,13-34].

## Methods

**Fig. 1.**
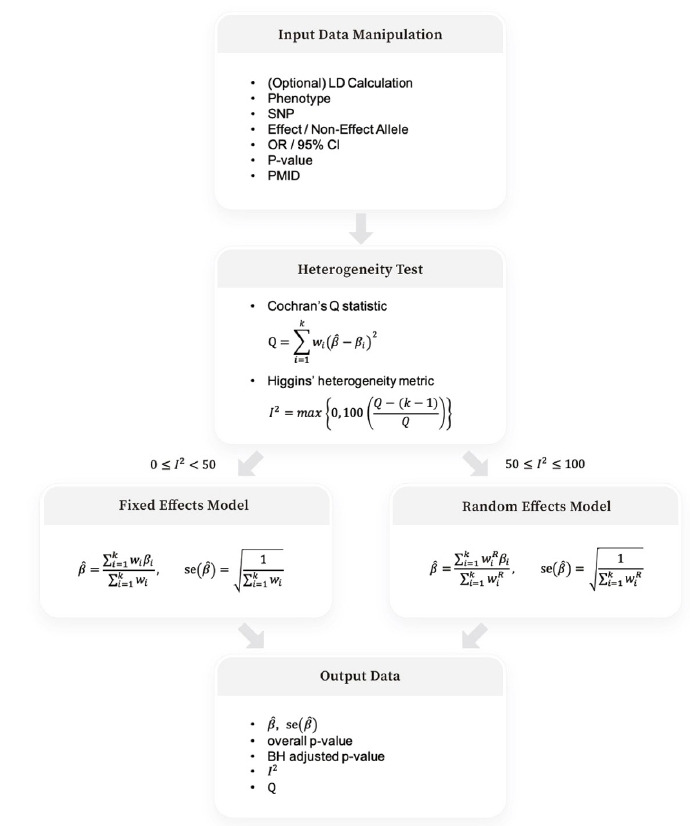
Overview of Beta-Meta pipeline.

Fig. 1 depicts the four steps of Beta-Meta: input data manipulation, heterogeneity test, weighted effect size calculation under the fixed and random effects models, and output data of summary statistics after the BH adjustment.

### Linkage disequilibrium calculation

Meta-analysis can improve signal detection when we account for not only between-study heterogeneity but also differences in linkage disequilibrium (LD) between ethnicities [35]; in addition, several trans-ethnic meta-analyses have identified unknown susceptibility genes [35-37]. As it is important to consider differences in LD, we utilize the haploR package [12] that queries HaploReg database [38] and returns alternative SNPs in LD. By calculating pairwise metrics of LD in each continental population, LD structures between ethnicities can be discovered and hence alternative SNPs can be used for the following meta-analysis [38]. This step is optional; users may skip this step and start a meta-analysis when the summary statistics of their target SNPs of interest are already obtained.

### Input data manipulation

After surveying the studies of interest (infertility-related disorders in this paper), we created a table for input data in Excel (Supplementary Table 1). Beta-Meta can read an Excel file for input data, which must include phenotypes, SNPs, effect and non-effect alleles, effect sizes, and p-values. For the effect sizes and their levels of significance, either the beta coefficient and its standard error or the odds ratio (OR) and its confidence interval can be used. As Beta-Meta calculates SNP-phenotype associations separately, it is acceptable to include as many phenotypes as desired in the single input file.

When the OR and its confidence interval are used for input data, they are converted into the beta coefficient and the standard error, respectively. The normalized effect of the *i^th^*study, *β_i_* is the logarithm of OR, where *k* is the number of individual studies, each of which is designed to examine the same SNP-phenotype association [9].


$$\beta_{i}=\ln OR_{i}(i=1,2,\cdots ,k)$$

The standard error *s_i_* is calculated from the 95% confidence interval of the OR.


$$s_{i}=\frac{\ln OR_{upper,i}-\ln OR_{lower,i}}{3.92}(i=1,2,\cdots ,k)$$

When synthesizing datasets for meta-analysis, it is important to ensure uniformity in allele labels and hence in the direction of the effect because alleles are typically called on only one of the two DNA strands in sequencing experiments [39]. Beta-Meta automatically corrects the direction of the effect by using one of the datasets with the lowest p-value as a reference and aligning the other datasets to it. For example, when the effect and the non-effect allele are inverted between the independent studies (e.g., rs13405782 and rs1801133 as shown in Table 1), this can be resolved automatically by changing the sign of the normalized effect.

### Heterogeneity analysis

In meta-analysis, datasets generated by multiple groups by different methods are likely to have any kind of variability, also known as heterogeneity. Heterogeneity indicates that the observed effects in datasets are more different from each other than would be expected by random error alone [40]. To check the heterogeneity, the weighted average of the effect size $\hat{\beta }$ is calculated first as [9]:


$$\hat{\beta }=\frac{\sum_{i=1}^{k}w_{i}\beta_{i}}{\sum_{i=1}^{k}w_{i}}\left(w_{i}=\frac{1}{s_{i}^{2}}\right)$$

Then, we calculate the Cochran’s Q statistic, Q and Higgins’ heterogeneity metric, *I^2^* for the heterogeneity test [6].


$$Q=\sum_{i=1}^{k}w_{i}{\left(\hat{\beta }-\beta_{i}\right)}^{2}$$

$$I^{2}=\max\left\{0,100(\frac{Q-(k-1)}{Q})\right\}$$

*I^2^* quantifies the degree of heterogeneity as a value between 0 and 100% [41]. As a greater value of *I^2^* indicates stronger heterogeneity, the weighted average of the effect sizes is calculated, based on *I^2^*, using two different models: the fixed effects model and the random effects model. A threshold value of *I^2^* for the model selection is set to 50%.

### Calculation of weighted average of the effect sizes based on *I^2^*

For 0≤*I^2^*<50, we use the fixed effects model to calculate the weighted average of the effect sizes and its standard error [7].


$$\hat{\beta }=\frac{\sum_{i=1}^{k}w_{i}\beta_{i}}{\sum_{i=1}^{k}w_{i}},se(\hat{\beta })=\sqrt{\frac{1}{\sum_{i=1}^{k}w_{i}}}$$

For 50≤I2≤100, we use the random effects model [7,9].


$$\hat{\beta }=\frac{\sum_{i=1}^{k}w_{{}_{i}}^{r}\beta_{i}}{\sum_{i=1}^{k}w_{{}_{i}}^{r}},se(\hat{\beta })=\sqrt{\frac{1}{\sum_{i=1}^{k}w_{{}_{i}}^{r}}}$$

The weights for the random effect model *w_i_^R^* are as follows [7,9]:


$$w_{i}^{R}=\frac{1}{\left(\frac{1}{w_{i}}+\tau^{2}\right)}(i=1,2,\cdots ,k)$$

$$where\;\tau^{2}=max\left\{0,\;\frac{\left(Q-\left(k-1\right)\right)}{\left(\sum_{i=1}^{k}w_{i}-\left(\frac{\sum_{i=1}^{k}w_{i}^{2}}{\sum_{i=1}^{k}w_{i}}\right)\right)}\right\}$$

### Integrated p-value and the BH adjustment

The integrated p-value through meta-analysis can be obtained as follows [7]:


p=2Φ(-|Z|)

where Φ is the cumulative distribution function of the standard normal distribution, and integrated *Z*-score, *Z* [7] is


$$Z=\frac{\overbrace{\beta }}{se\left(\overbrace{\beta }\right)}$$

Finally, to reduce the false-positive results, the integrated p-values are corrected by the BH adjustment method. When *p*_(1)_, *p*_(2)_, ⋯,*p*_(m)_ are the p-values of the SNPs sorted in ascending order (*p*_(1)_ ≤ *p*_(2)_ ≤ ⋯ ≤ *p*_(m)_), the adjusted p-values obtained through the BH procedure are as follows [11]:


$$p_{\left(j\right)}^{'}=\frac{m}{j}p\left(j\right)\left(j=1,\cdots .m\right)$$

where *m* is the number of different SNPs related to a specific phenotype, and *j* is the ranking in the ascending order of the p-values of SNPs related to the specific phenotype.

## Results

Using Beta-Meta, we performed a sample test of integrating multiple studies of infertility and obtained a table containing all of the above calculated summary statistics values (Supplementary Table 2) and a forest plot of combined effect sizes (Supplementary Fig. 1). The conventional genome-wide significance p-value threshold of 5 × 10^-8^ was used to identify significant SNP markers. Of the total 26 SNP-phenotype associations from the 23 studies we investigated (Supplementary Table 1), the only significant association was the one between rs10965232 and endometriosis from Uno et al. [14] with a p-value of 5.57 × 10^-12^ (Table 1). After performing the meta-analysis, we found three more significantly associated SNPs: rs13405728, rs1801133, and rs10842262 as displayed in Table 2.

In order to check the accuracy of Beta-Meta, we compared the meta-analysis results of Beta-Meta (Supplementary Table 2) with those of METAL [8] (Supplementary Table 3), which is one of the most widely used meta-analysis packages but does not have a random effects option. We could confirm the accuracy of Beta-Meta calculation with the result that the significantly associated SNPs identified by METAL and those found by Beta-Meta were the same. At the same time, Beta-Meta features convenience as it calculates the summary statistics accurately by automatically selecting the appropriate model based on heterogeneity.

## Discussion

Beta-Meta application can be utilized as an effortless meta-analysis tool for researchers with limited statistics backgrounds. It allows them to easily manipulate and analyze their own datasets on a personal computer as it is written in python and can be run with an executable file in MS Windows.

As shown above, Beta-Meta increases the power to detect weak signals, identifying significant variants which was not significantly associated in single studies. Furthermore, it calculates the effect sizes and the p-values accurately by selecting the appropriate model based on heterogeneity and applying the BH adjustment. These can contribute to time-efficient management of the recent growth in aggregated GWAS especially for those involved in the field of genetic testing. Because it is difficult to obtain a large number of datasets and validate genotype-phenotype associations experimentally within a limited budget, meta-analysis is still in demand to discover SNP markers for genetic testing.

In conclusion, the application presented here provides a conventional and yet convenient way to conduct a meta-analysis of GWAS. Beta-Meta is expected to facilitate various research projects, such as the discovery of novel SNP markers, the calculation of polygenic risk scores, and the acquisition of biological insights into complex diseases and traits.

## Figures and Tables

**Table 1.** Example of input data: summary statistics of the individual GWAS of infertility

Phenotype	SNP	EA	NEA	OR (95% CI)	p-value	PMID
Endometriosis	rs10965235	C	A	1.489	1.30E-4	25154675
				(1.213–1.827)		
Endometriosis	rs10965235	C	A	1.44	5.57E-12	20601957
				(1.3–1.59)		
Polycystic ovary syndrome	rs13405728	A	G	1.55	1.00E-03	34403018
				(1.39–1.72)		
Polycystic ovary syndrome	rs13405728	G	A	0.723	1.00E-03	30182769
				(0.686–0.762)		
Folic acid metabolism-related male infertility	rs1801133	T	C	1.33	1.40E-02	16247718
				(1.06–1.66)		
Folic acid metabolism-related male infertility	rs1801133	C	T	0.7	1.00E-05	30813130
				(0.66–0.75)		
Non-obstructive azoospermia	rs10842262	G	C	1.335	2.30E-03	24648396
				(1.1081–1.6083)		
Non-obstructive azoospermia	rs10842262	G	C	1.23	0.001	30863997
				(1.16–1.3)		

GWAS, genome-wide association studies; SNP, single nucleotide polymorphism; OR, EA, effect allele; NA, non-effect allele; odds ratio; CI, confidence interval.

**Table 2.** Example of output data presenting only the significantly associated SNPs after meta-analysis

Phenotype	SNP	EA	NEA	$\hat{\beta }$	se( $\hat{\beta }$)	p-value	adjusted p-value	I^2^	Q
Endometriosis	rs10965235	C	A	0.371	0.046	8.2E-16	8.2E-16	0	0.083
Polycystic ovary syndrome	rs13405728^a^	A	G	0.371	0.056	3.55E-11	7.11E-11	71.70	3.534
Folic acid metabolism-related male infertility	rs1801133	C	T	-0.351	0.031	4E-29	8E-29	0	0.361
Non-obstructive azoospermia	rs10842262	G	C	0.214	0.028	1.36E-14	1.36E-14	0	0.679

SNP, single nucleotide polymorphism; EA, effect allele; NEA, non-effect allele.

a For rs13405728, the integrated effect size and p-value were calculated under the random effects model as its I^2^ was greater than 50.

## Supplementary Materials

Supplementary Table 1. Input data: summary statistics of the individual GWAS of infertility

Supplementary Table 2. Output data: summary statistics after meta-analysis

Supplementary Table 3. Output data: summary statistics after meta-analysis using METAL

Supplementary Fig. 1. Forest plot of the combined effect sizes. Forest plot of 95% confidence intervals of the combined effect sizes after meta-analysis.
