# Supplementary material for: Beta-Meta: a meta-analysis application considering heterogeneity among genome-wide association studies
Source: Genomics Inform. 2022 Dec 30;20(4):e49. doi: 10.5808/gi.22046 (PMC9847376; doi:10.5808/gi.22046)
Supplement: Supplementary Table 1. — Input data: summary statistics of the individual GWAS of infertility [file gi-22046suppl1.pdf]

Supplementary Table 1. Input data: summary statistics of the individual GWAS of infertility

Phenotype	SNP	Effect_Allele	Non_Effect_Allele	Beta	Beta_SE	OR	OR_95% CI_Lower	OR_95% CI_Upper	p-value	PMID
Endometriosis	rs10965235	C	A			1.489	1.213	1.827	1.30E-04	25154675
Endometriosis	rs10965235	C	A			1.44	1.3	1.59	5.57E-12	20601957
Polycystic ovary syndrome	rs6166	G	A			1.87	1.14	3.06	1.30E-02	28547204
Polycystic ovary syndrome	rs6166	A	G			0.72	0.6	0.87	1.00E-03	34403018
Polycystic ovary syndrome	rs13405728	A	G			1.55	1.39	1.72	1.00E-03	34403018
Polycystic ovary syndrome	rs13405728	G	A			0.723	0.686	0.762	1.00E-03	30182769
Recurrent spontaneous abortion	rs1799983	T	G			2.39	1.25	4.58	8.00E-03	20728041
Recurrent spontaneous abortion	rs1799983	T	G			1.585	1.175	2.138	5.00E-02	30681586
Recurrent spontaneous abortion	rs11614913	C	T			1.987	1.227	3.217	2.00E-02	22222140
Recurrent spontaneous abortion	rs11614913	C	T			1.828	1.145	2.921	1.20E-02	22882355
Recurrent spontaneous abortion	rs3025039	T	C			1.26	1.04	1.53	2.00E-02	28282525
Recurrent spontaneous abortion	rs3025039	T	C			1.6492	1.2023	2.2622	1.80E-03	23211130
DNA damage-related male infertility	rs25487	G	A			0.614	0.4	0.937	2.40E-02	32358378
DNA damage-related male infertility	rs25487	A	G			1.183	1.057	1.325	3.00E-03	32535968
Folic acid metabolism-related male infertility	rs1801133	T	C			1.33	1.06	1.66	1.40E-02	16247718
Folic acid metabolism-related male infertility	rs1801133	C	T			0.7	0.66	0.75	1.00E-05	30813130
Folic acid metabolism-related male infertility	rs1801394	A	G			0.76	0.66	0.88	3.00E-05	30813130

Folic acid metabolism-related male infertility	rs1801394	G	A	1.78	1.01	3.17	4.69E-02	16861746
Male infertility due to oxidative stress	rs2070744	C	T	1.41	1.11	1.78	4.00E-03	27373555
Male infertility due to oxidative stress	rs2070744	C	T	1.4	1.08	1.82	1.00E-02	25505202
Oligoasthenoteratozoospermia	rs366631	A	G	1.53	1.25	1.86	0.001	23877133
Oligoasthenoteratozoospermia	rs366631	A	G	1.69	1.22	2.32	1.00E-03	23026209
Oligoasthenoteratozoospermia	rs1801133	T	C	1.7	1.08	2.69	2.09E-02	16247718
Oligoasthenoteratozoospermia	rs1801133	T	C	1.35	1.2	1.52	0.001	33371103
Non-obstructive azoospermia	rs10842262	G	C	1.335	1.1081	1.6083	2.30E-03	24648396
Non-obstructive azoospermia	rs10842262	G	C	1.23	1.16	1.3	0.001	30863997

GWAS, genome-wide association studies; SNP, single nucleotide polymorphism; SE, standard error; OR, odds ratio; CI, confidence interval.
