# Supplementary material for: Beta-Meta: a meta-analysis application considering heterogeneity among genome-wide association studies
Source: Genomics Inform. 2022 Dec 30;20(4):e49. doi: 10.5808/gi.22046 (PMC9847376; doi:10.5808/gi.22046)
Supplement: Supplementary Table 2. — Output data: summary statistics after meta-analysis [file gi-22046suppl2.pdf]

Supplementary Table 2. Output data: summary statistics after meta-analysis

Phenotype	SNP	Effect_Allele	Non_Effect_Allele	Beta	Beta_SE	p-value	BH_p-value	I_Square	Q_HET
Endometriosis	rs10965235	C	A	0.371156955	0.046099499	8.19765E-16	8.19765E-16	0	0.082597717
Polycystic ovary syndrome	rs6166	A	G	-0.365398885	0.088713211	3.80722E-05	3.80722E-05	18.12758975	1.22141268
Polycystic ovary syndrome	rs13405728	A	G	0.371491629	0.056103423	3.55451E-11	7.10903E-11	71.70403437	3.534072712
Recurrent spontaneous abortion	rs1799983	T	G	0.532563967	0.138679394	0.000122908	0.000368723	21.12092753	1.267763386
Recurrent spontaneous abortion	rs11614913	C	T	0.643723897	0.171347836	0.000172082	0.000258124	0	0.059182221
Recurrent spontaneous abortion	rs3025039	T	C	0.335417977	0.13113816	0.010535331	0.010535331	50.72911937	2.02959636
DNA damage-related male infertility	rs25487	A	G	0.259359975	0.144410574	0.072496076	0.072496076	50.61625676	2.02495788
Folic acid metabolism-related male infertility	rs1801133	C	T	-0.351304175	0.031361794	4.00162E-29	8.00323E-29	0	0.36107873
Folic acid metabolism-related male infertility	rs1801394	A	G	-0.292415627	0.071171592	3.98064E-05	3.98064E-05	0.863749625	1.008712753
Male infertility due to oxidative stress	rs2070744	C	T	0.340385357	0.089328047	0.000138681	0.000138681	0	0.001571401
Oligoasthenoteratozoospermia	rs366631	A	G	0.452779295	0.086231296	1.51478E-07	1.51478E-07	0	0.266202151
Oligoasthenoteratozoospermia	rs1801133	T	C	0.314599743	0.058376569	7.0794E-08	1.41588E-07	0	0.918875485
Non-obstructive azoospermia	rs10842262	G	C	0.214022183	0.027796259	1.36412E-14	1.36412E-14	0	0.679449034

SNP, single nucleotide polymorphism; SE, standard error.
