# Supplementary material for: Beta-Meta: a meta-analysis application considering heterogeneity among genome-wide association studies
Source: Genomics Inform. 2022 Dec 30;20(4):e49. doi: 10.5808/gi.22046 (PMC9847376; doi:10.5808/gi.22046)
Supplement: Supplementary Table 3. — Output data: summary statistics after meta-analysis using METAL [file gi-22046suppl3.pdf]

Supplementary Table 3. Output data: summary statistics after meta-analysis using METAL

MarkerName	Allele1	Allele2	Effect	StdErr	P-value	Direction
rs10965235	a	c	-0.3712	0.0461	8.198e-16	--
rs6166	a	g	-0.3654	0.0887	3.807e-05	--
rs13405728*	a	g	0.3466	0.0240	3.867e-47	++
rs1799983	t	g	0.5326	0.1387	0.0001229	++
rs11614913	t	c	-0.6437	0.1713	0.0001721	--
rs3025039*	t	c	0.3042	0.0840	0.0002947	++
rs25487*	a	g	0.1891	0.0557	0.0006889	++
rs1801133[a]	t	c	0.3513	0.0314	4.002e-29	++
rs1801394	a	g	-0.2924	0.0712	3.981e-05	--
rs2070744	t	c	-0.3404	0.0893	0.0001387	--
rs366631	a	g	0.4528	0.0862	1.515e-07	++
rs1801133[b]	t	c	0.3146	0.0584	7.079e-08	++
rs10842262	c	g	-0.2140	0.0278	1.364e-14	--

To compare the meta-analysis results of Beta-Meta (Supplementary Table 2) with those of METAL, the same input data (Supplementary Table 1) was used for the both analyses.

*The integrated effect sizes and p-values are different from the results of Beta-Meta because METAL uses the fixed effects model only, whereas Beta-Meta applies the random effects model to the set of studies in which significant heterogeneity is present (I^2 > 50).

[a] Association between rs1801133 and folic acid metabolism-related male infertility.

[b] Association between rs1801133 and oligoasthenoteratozoospermia.
