# Supplementary material for: Beta-Meta: a meta-analysis application considering heterogeneity among genome-wide association studies
Source: Genomics Inform. 2022 Dec 30;20(4):e49. doi: 10.5808/gi.22046 (PMC9847376; doi:10.5808/gi.22046)
Supplement: Supplementary Fig. 1. — Forest plot of the combined effect sizes. Forest plot of 95% confidence intervals of the combined effect sizes after meta-analysis. [file gi-22046suppl4.pdf]

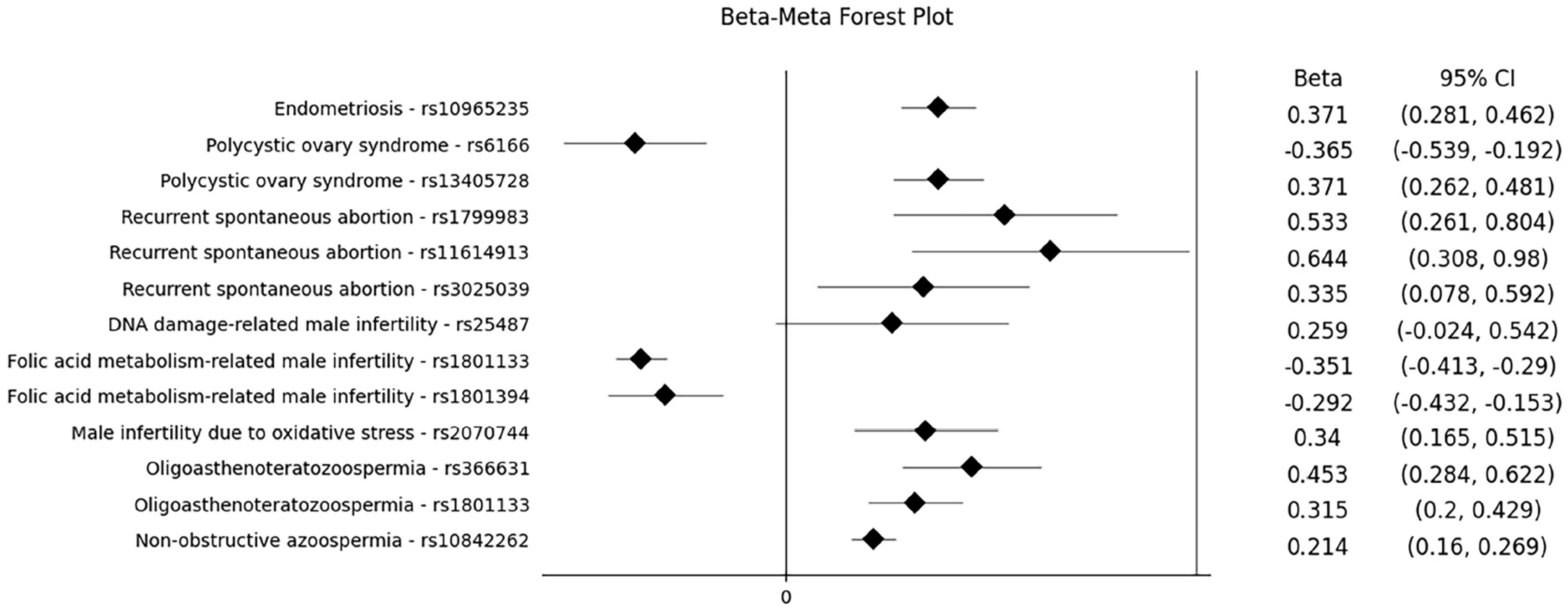

Supplementary Fig. 1. Forest plot of the combined effect sizes. Forest plot of 95% confidence intervals of the combined effect sizes after meta-analysis.
